# MEDI: Macronutrient Extraction and Determination from invertebrates, a rapid, cheap and streamlined protocol

**DOI:** 10.1111/2041-210X.13551

**Published:** 2021-01-22

**Authors:** Jordan P. Cuff, Shawn M. Wilder, Maximillian P. T. G. Tercel, Rhiannon Hunt, Somoye Oluwaseun, Paige S. Morley, Rafael A. Badell‐Grau, Ian P. Vaughan, James R. Bell, Pablo Orozco‐terWengel, William O. C. Symondson, Carsten T. Müller

**Affiliations:** ^1^ School of Biosciences Cardiff University Cardiff UK; ^2^ Rothamsted Research Harpenden UK; ^3^ Department of Integrative Biology Oklahoma State University Stillwater OK USA

**Keywords:** arthropod, carbohydrate, colorimetric, exoskeleton, lipid, protein

## Abstract

1. Macronutrients, comprising carbohydrates, proteins and lipids, underpin many ecological processes, but their quantification in ecological studies is often inaccurate and laborious, requiring large investments of time and bulk samples, which make individual‐level studies impossible. This study presents Macronutrient Extraction and Determination from Invertebrates (MEDI), a protocol for the direct, rapid and relatively low‐cost determination of macronutrient content from single small macroinvertebrates.
2. Macronutrients were extracted by a sequential process of soaking in 1:12 chloroform:methanol solution to remove lipid and then solubilising tissue in 0.1 M NaOH. Proteins, carbohydrates and lipids were determined by colorimetric assays from the same individual specimens.
3. The limits of detection of MEDI with the equipment and conditions used were 0.067, 0.065 and 0.006 mg/ml for proteins, carbohydrates and lipids respectively. Adjusting the volume of reagents used for extraction and determination can broaden the range of concentrations that can be detected. MEDI successfully identified taxonomic differences in macronutrient content between five insect species.
4. Macronutrient Extraction and Determination from Invertebrates can directly and rapidly determine macronutrient content in tiny (dry mass ~3 mg) and much larger individual invertebrates. Using MEDI, the total macronutrient content of over 50 macroinvertebrates can be determined within around 3 days of collection at a cost of ~$1.35 per sample.

## Abstract (cy)

1. Mae macromaetholion, sy'n cynnwys carbohydradau, proteinau a lipidau, yn sail i lawer o brosesau ecolegol, ond mae eu meintioli mewn astudiaethau yn aml yn medru bod yn wallus ac yn llafurus, ac yn galw am fuddsoddiadau mawr mewn amser a swmp‐samplau. Gwna hyn astudiaethau ar lefel unigol yn amhosibl. Mae'r astudiaeth hon yn disgrifio MEDI (Echdyniad ac Adnabod Macromaetholion o Anifeiliaid Di‐asgwrn‐cefn), protocol newydd sy'n mesur ac adnabod macromaetholion yn uniongyrchol, ond yn gyflym ac yn gymharol rad, o facroinfertebratau bach sengl.
2. Echdynnwyd macromaetholion trwy glain‐guriad mewn hydoddiant clorofform 1:12 methanol i waredu'r lipid, cyn hydoddi'r meinwe mewn 0.1M NaOH. Adnabuwyd proteinau, carbohydradau a lipidau gan brofion lliwmetrig o'r un sbesimenau.
3. Cyfyngiadau darganfod MEDI gyda'r offer a ddefnyddiwyd oedd 0.067, 0.065 a 0.006 mg/ml ar gyfer proteinau, carbohydradau a lipidau, yn ôl eu trefn. Gall addasu cryfderau'r adweithyddion a ddefnyddir i echdynnu ac adnabod ehangu'r ystod o grynodiadau y gellir eu canfod. Llwyddodd MEDI i nodi gwahaniaethau tacsonomig mewn cynnwys macrofaetholion rhwng pum rhywogaeth o bryfed.
4. Gall MEDI bennu cynnwys macromaetholion yn gywir, yn uniongyrchol ac yn gyflym, mewn anifeiliaid di‐asgwrn cefn bach (màs sych ~3 mg) a mawr. Gan ddefnyddio MEDI, gellir pennu cyfanswm cynnwys dros 50 o facromaetholion o fewn oddeutu tridiau am gost o tua $ 1.35 y sampl.

## 1 INTRODUCTION

Despite the relevance of macronutrients, comprising proteins, carbohydrates and lipids, to a broad range of applications, few ecological studies quantify them. Many studies concerned with the macronutrient content of invertebrates use analogues, such as nitrogen as a surrogate for protein (e.g. crude protein = N × 6.25; Jones, 1931) or in lieu of protein (Bryer et al., 2015; Finke, 2005; Pekár & Mayntz, 2014). This allows broad‐scale studies of ecological stoichiometry in trophic networks, focusing on the ratios of analogous elements such as carbon, nitrogen and phosphorous (Anderson & Hessen, 2005; Frost et al., 2005; Raubenheimer et al., 2009). While broadly useful, these analogues can produce inaccurate results since, for example, nitrogen is present in many non‐protein constituents of invertebrates, including exoskeleton (Janssen et al., 2017; Jones, 1931; Raubenheimer et al., 2009). Correction factors may circumvent these issues, but one correction factor is unlikely to work on all species given the diversity of invertebrates (Janssen et al., 2017). Additionally, some analyses of macronutrient content use gravimetric methods (e.g. Pekár & Mayntz, 2014), which require either bulk samples (~1 kg insect material for Finke, 2013) or very fine, often expensive, scales for the determination of macronutrient mass, long waiting times, and often still rely on analogues. Bulk samples are laborious to collect and process, impeding multi‐taxon or individual‐level analyses (Bryer et al., 2015).

Methods have previously been developed for determining the macronutrient content of small single macroinvertebrate samples (e.g. Lu et al., 2008), but these are standalone protocols each tailored to only one macronutrient, tripling the collection effort necessary to determine the content of each macronutrient from a population and making individual‐level studies impractical. By implementing a uniform extraction method and streamlining a protocol to determine all three macronutrient contents from a single specimen, information output would increase while reducing sampling effort. No protocol has yet been published which uses direct measures of all three macronutrients taken in parallel from single small invertebrate specimens. Standardised adoption of such a protocol would also ultimately benefit future meta‐analyses. For individual‐level determination of macronutrient content, or studies involving particularly small or scarce invertebrates, there is a need for a standardised approach to directly determine macronutrient content in parallel from single macroinvertebrate specimens to better understand ecological nutrient dynamics.

Our protocol determines the content of all three macronutrients from the same individual specimen. Presented herein is Macronutrient Extraction and Determination from Invertebrates (MEDI), a streamlined, rapid, cheap and simple protocol for the extraction and determination of macronutrient content that can be applied at the scale of individual invertebrates (≥~3 mg dry mass). Using MEDI, the total carbohydrate, lipid and protein content of over 50 macroinvertebrates can be determined within around 3 days of collection at a cost of ~$1.35 per sample using standard laboratory equipment. This protocol will enhance the study of macronutrient content in invertebrates and other small samples in contexts including trophic interactions, parasitology and development.

## 2 DESCRIPTION AND IMPLEMENTATION

### 2.1 Materials

All materials, unless stated otherwise, were purchased from Sigma‐Aldrich. Flat bottom, 96‐well microplates (Sterilin Microplate F Well), Pierce BCA Protein Assay reagents and Pierce Modified Lowry Protein Assay reagents were obtained from Thermo Fisher Scientific. Ribbed, skirted 1.5 ml screwcap microtubes and caps were obtained from STARLAB. Sulphuric acid (95%) and phosphoric acid (85%) were obtained from Fisher Scientific.

### 2.2 Macronutrient extraction

**FIGURE 1:**
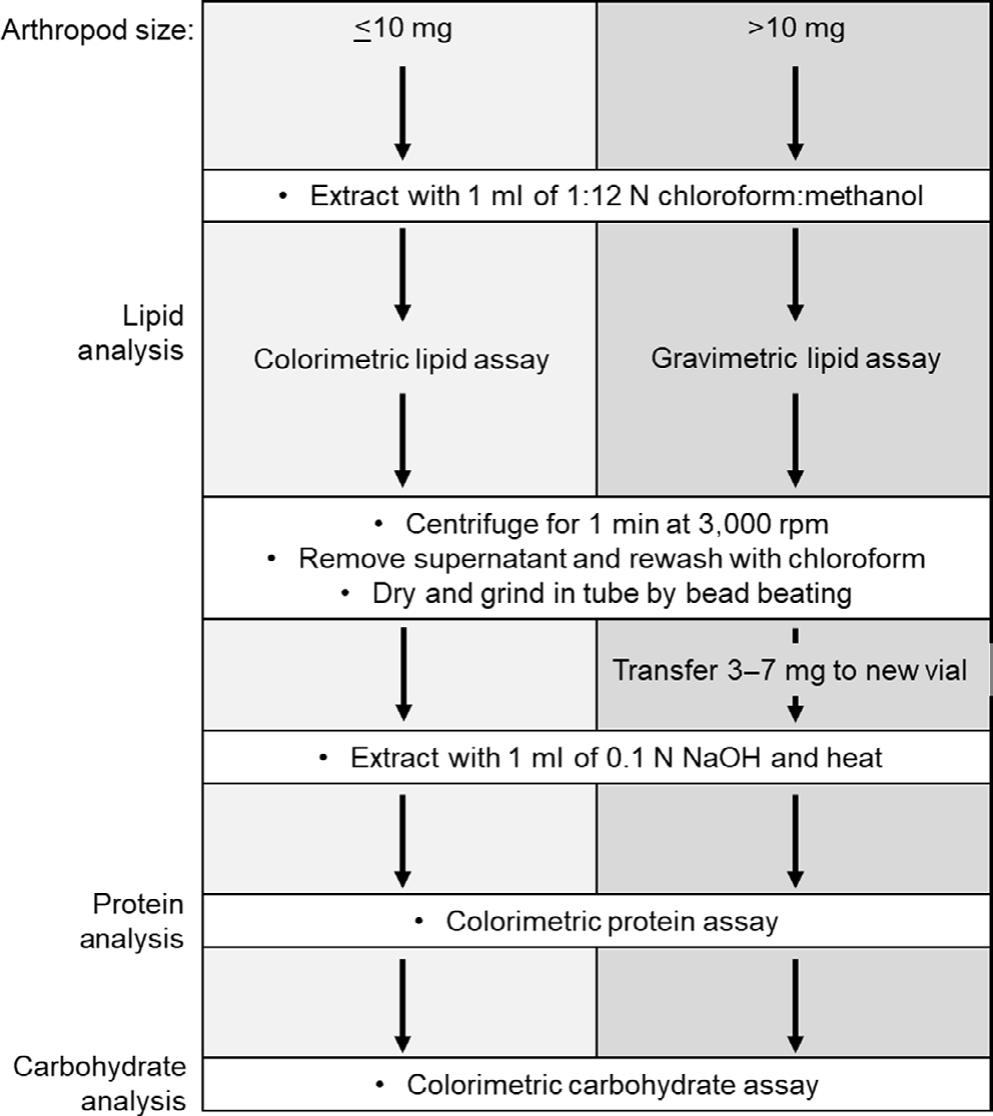
Workflow of MEDI for specimens of different sizes

**FIGURE 2:**
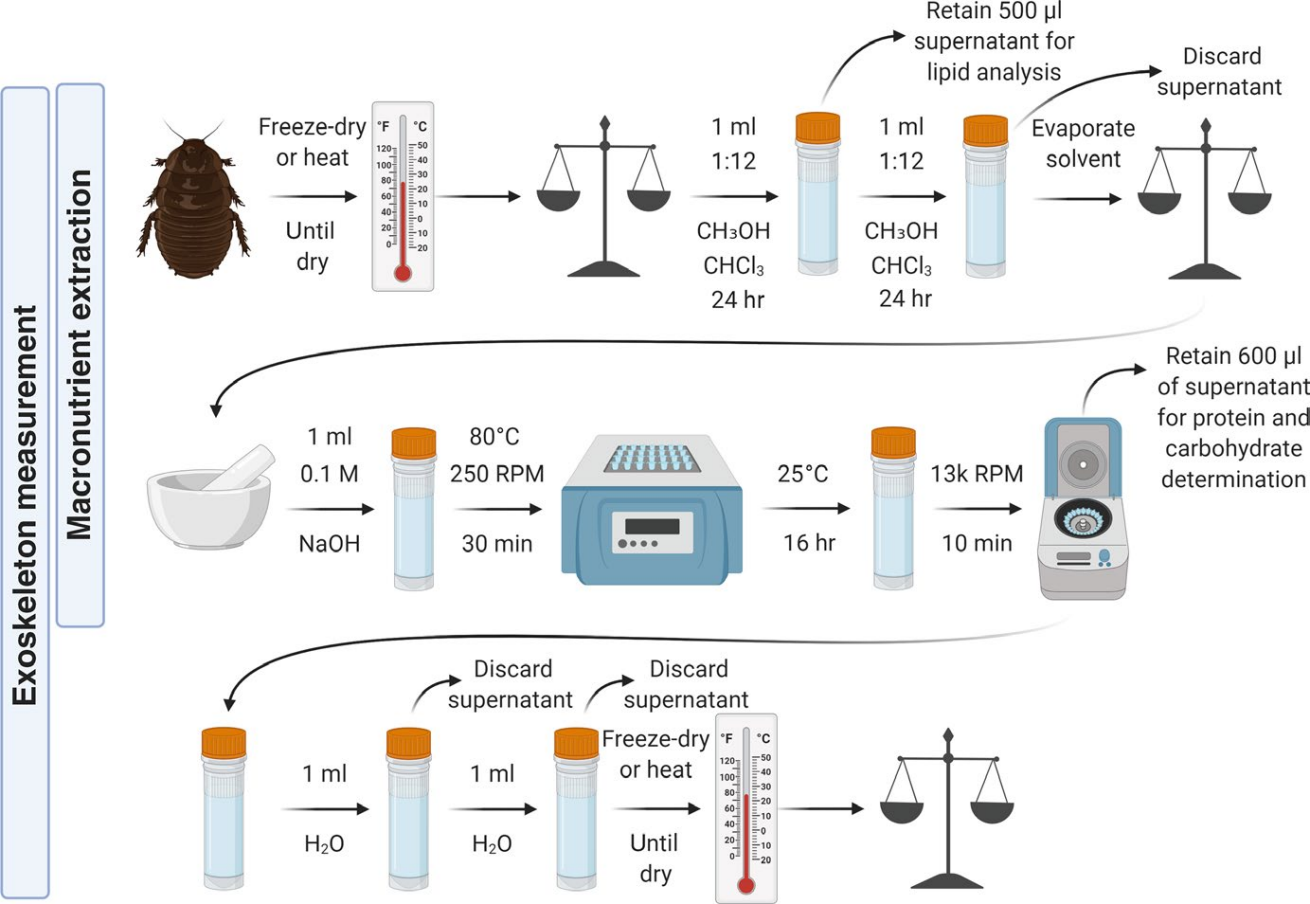
Protocol for the extraction of macronutrients and measurement of exoskeletal mass from invertebrate bodies. Only the first two rows are carried out for macronutrient extraction. For exoskeletal measurement, separate samples were used in this study. It is advised to lyse the specimen for protein extraction because this allows efficient extraction of protein in one wash of NaOH. But it is not advised to lyse the specimen for exoskeletal measurement because preliminary work suggests that exoskeleton measurements on lysed samples are significantly lower than measurements on intact samples (S.M. Wilder, unpubl.); it is also advised to heat the specimen for a longer period (i.e. 2 hr) and to repeat the central steps (addition of NaOH, heating, incubation and discarding of supernatant) to ensure removal of all soft tissues. Figure created using Biorender.com

Macronutrient extraction is a two‐step process that first involves extracting lipid and then solubilising the remaining tissue for carbohydrate and protein analysis (Figures 1 and 2). Details of the methods will vary depending on the size of arthropod used. There are many important considerations when analysing the macronutrient content of arthropods (Table 1).

**TABLE 1:** Considerations when analysing the macronutrient content of arthropod samples

Technique	Options	Information	Best practice suggestion
Protein assay	Crude Protein (6.25 × % nitrogen)	Assumes that all nitrogen in a sample is in the form of protein with 16% nitrogen	The estimated protein content of a sample will vary depending on the method used and each has biases. Ideally, analysis of hydrolysed amino acids could be used to determine which assay is most appropriate for a group of organisms. Alternatively, users can measure samples using multiple assays and take the average of those estimates
	Bradford	Primarily reacts with arginine, lysine and histidine	
	BCA	Primarily reacts with cysteine/cystine, tyrosine and tryptophan	
	Lowry	Primarily reacts with cysteine/cystine, tyrosine and tryptophan	
	Hydrolysed Amino Acid Analysis	Considered one of the most accurate measures of protein and provides measures of amino acid composition of samples but is far more expensive	
Protein standard	Bovine serum albumin (BSA) versus immunoglobulin G (IgG) versus bovine gamma globulin (BGG)	Protein standards differ in amino acid content. Given that protein assays primarily react with only several amino acids, the choice of protein standard will affect the estimate of protein measured with the assay	Most protein assays note conversion factors that can be used to convert protein measures estimated with one standard to an estimate based on another standard. Users could take the average of the estimate from BSA and IgG rather than choosing to present data based on one or the other standard
Lipid assay	Colorimetric	Some will only, or primarily, measure certain types of lipids (e.g. the sulfo‐phospho‐vanillin assay only detects unsaturated lipids)	First consider the size of the invertebrate. Colorimetric assays are the most practical solution for very small invertebrates (e.g. <5 mg dry mass). Then consider what lipids you want to measure to address the goals of your study (e.g. a specific type or all lipids)
		May be better for life‐history studies in which users are interested in measuring specific types of lipids	
		May be used on any size of invertebrate, including individual collembolans or aphids	
	Gravimetric	Measures total lipid content, which can include triglycerides and phospholipids. This is a very easy assay, especially on larger invertebrates. This can be a better measure of nutrients available to consumers of an arthropod	
Carbohydrate assay	Simple sugars	Not a common form of carbohydrate in insects, mainly found in sap or nectar feeding insects. Choice of standard (e.g. glucose vs. sucrose) may be important	The user must consider the goals of the study, particularly the reason for measuring carbohydrates and which carbohydrates are most relevant to addressing the study question. The anthrone assay will detect simple sugars and will break down glycogen and trehalose, but other assays could be considered on a case‐by‐case basis for further applications
	Glycogen and Trehalose	These are common forms in which carbohydrates are stored in insects	
Exoskeleton determination		This assay measures the mass of exoskeleton present in an arthropod	This may be useful to measure in studies of arthropod morphology or when measuring the quality of arthropods as food for predators since exoskeletal chitin is indigestible to most consumers and is equally unassimilated by predators with extra‐oral digestion

The aphid *Metopolophium dirhodum* (Walker, 1849; Hemiptera: Aphididae), house cricket *Acheta domesticus* (Linnaeus, 1758; Orthoptera: Gryllidae), German cockroach *Blattella germanica* Linnaeus, 1767 (Blattodea: Ectobiidae), mealworm larvae *Tenebrio molitor* Linnaeus, 1758 (Coleoptera: Tenebrionidae) and springtail *Folsomia candida* Willem, 1902 (Entomobryomorpha: Isotomidae) were used to test the protocol's limits of detection, given their ease of cultivation and range of dry masses (in this study, mean ± *SD*, *F. candida* 1.14 ± 0.55 mg, *M. dirhodum* 3.10 ± 0.65 mg, *A. domesticus* 22.20 ± 5.83 mg, *B. germanica* 22.53 ± 4.96 mg, *T. molitor* 36.20 ± 22.30).

Samples were first weighed and lipids were extracted by soaking whole arthropods in 1 ml of 1:12 chloroform:methanol for 24 hr (smaller specimens such as those <0.5 mg dry mass could be soaked in 0.5 ml for increased detectability, and larger specimens in larger volumes ~5× their body volume to ensure full submersion and to prevent saturation of the solvent). Half of the added volume of supernatant was then pipetted into a fresh tube for later lipid determination, the rest of the supernatant discarded, and any residue allowed to evaporate. This procedure for soaking arthropods was repeated for another 24 hr, but discarding all supernatant, to ensure any residual lipids were removed from the sample prior to protein and carbohydrate extraction. The change in dry mass of a sample before and after soaking in the solvent can also be used as an estimate of the lipid content of samples where practicable (i.e. gravimetric assay).

Following the lipid assay, the soft tissue of samples was digested to facilitate quantification of protein and carbohydrates. This procedure only measures the macronutrient content of the soft tissue of arthropods and not any protein that may be bound in the chitinous matrix of the exoskeleton during sclerotisation. Whole arthropods from 1 to 10 mg lean mass (i.e. mass after lipid extraction) were weighed, added to a microcentrifuge tube along with a stainless‐steel bead (~3–7 mm diameter) and lysed at room temperature using a TissueLyser II (Qiagen) for 8 min at 30 Hz in 2‐min increments. Larger samples were ground (e.g. bead beating or mortar and pestle) and an approximately 5 mg subsample was weighed into a clean tube. To each tube was added 1 ml of 0.1 M NaOH (or 0.5 ml for smaller specimens, e.g. <1 mg). Tubes were placed in a thermo‐shaker at 80°C and 250 RPM for 30 min, then removed and left at room temperature overnight (~16 hr). Samples were centrifuged for 10 min at 13,000 rpm and 600 µl of supernatant pipetted into a separate tube for protein and carbohydrate determination. Supernatant was diluted prior to assaying such that the concentration of lean tissue (approximately 25%–75% protein for arthropods) was approximately 1–2 mg/ml to allow protein values to fall within the range of the protein assay kit (most commercial protein assay kits can measure 0.025–2 mg/ml protein). Dilution of supernatant or change in volume of NaOH used, along with the mass of sample used, must be accounted for in subsequent calculations of protein content.

### 2.3 Exoskeletal mass determination

The exoskeleton content of samples can also be measured, which may be of interest in morphological studies or those concerned with the nutritional quality of arthropods for consumers (Figure 2). A separate sample was used for this measurement in this study since lysis of tissues was carried out during macronutrient extraction to facilitate rapid dissolution of all soft tissues. Preliminary work suggests that exoskeleton measurements of lysed tissue result in lower values than measurements on intact arthropod bodies (S.M. Wilder, unpubl.). To maintain intact exoskeletons, the exoskeletal measurements instead included a second round of NaOH treatment and longer heated incubations. Exoskeletal measurement could theoretically be carried out on the same specimens used for macronutrient determination, but appropriate care must be taken to ensure that the soft tissue is appropriately dissolved; separate specimens should thus be used where possible. First, lipid should be completely extracted from the sample as described above. Then, the exoskeleton of the sample should be lightly cracked and 0.1 M NaOH (a volume approximately 5–10 times that of the sample) should be added to a vial with the sample. Samples should be heated for 2 hr at 80°C and then allowed to soak overnight after which the NaOH should be removed and discarded. Centrifugation may help move the exoskeleton to the bottom of the vial. An additional volume of NaOH is added to the tubes and allowed to soak for 24 hr at room temperature, after which the NaOH can again be removed and discarded. Similar volumes of water should then be added to samples and removed twice to rinse any remaining NaOH from samples. Exoskeleton content is then the mass of sample remaining in the vial.

### 2.4 Macronutrient determination

Colorimetric assays were selected for the determination of macronutrients, given their ease‐of‐use and capacity for high‐throughput assaying of samples in 96‐well plates (Cheng et al., 2011; Rodrı et al., 2008). All absorbance measurements were obtained from a Tecan Infinity M200 Pro plate reader (Tecan Life Sciences) with Magellan v.7.1 software. For all assays, standard dilution series for calibration of absorbance readings consisted of 0–2 mg/ml in nine increments (0, 0.025, 0.125, 0.25, 0.5, 0.75, 1, 1.5 and 2 mg/ml), with corn starch diluted in water, lard oil diluted in methanol and bovine serum albumin (BSA) diluted in water for carbohydrates, lipids and proteins respectively. For each assay three repeats were taken from each sample and standard.

**FIGURE 3:**
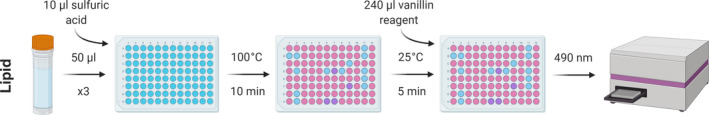
Protocol for the determination of lipid content using the sulfo‐phospho‐vanillin method (Supporting Information 1). Figure created using Biorender.com

For determination of lipids, a sulfo‐phospho‐vanillin method adapted from Cheng et al. (2011) was used (Figure 3, Supporting Information 1). This method determines unsaturated lipid content; for total lipid content, gravimetric methods are the most appropriate option, but difficult for small invertebrates without specialised scales. Samples for lipid analysis comprised the initial supernatant taken after chloroform/methanol extraction.

**FIGURE 4:**
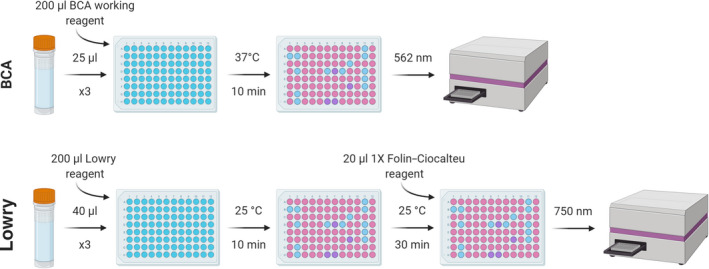
Protocol for the determination of protein content using the BCA and Lowry methods (Supporting Information 2). Figure created using Biorender.com

Given the range of available protein assays, each with different benefits, the same samples from the five species analysed were put through two different protein‐based colorimetric assays: bicinchoninic acid (BCA) and Lowry assays (Figure 4; Supporting Information 2). These assays followed the manufacturer protocols for BCA and Lowry assays. Samples for protein analysis comprised the supernatant taken after NaOH extraction.

**FIGURE 5:**
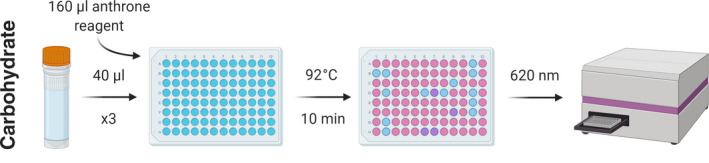
Protocol for the determination of carbohydrate content using the anthrone method (Supporting Information 3). Figure created using Biorender.com

For carbohydrate determination, the anthrone method, originally proposed by Dreywood (1946), was adapted (Figure 5; Supporting Information 3). Samples for carbohydrate analysis comprised the final supernatant taken after NaOH extraction.

For each assay, the absorbance measurement of the blank standard was subtracted from all other absorbance measurements and a standard calibration curve prepared by plotting the blank‐corrected measurement of the standards against their known concentrations. The regression equation of the standard curve was used to determine macronutrient concentration in each sample in mg/ml, which was then used to calculate the total macronutrient concentration in the sample based on the sample weight used for analysis and any dilution that was applied to the sample.

### 2.5 Limits of MEDI

Limit of blank (LoB) and limit of detection (LoD) describe the largest apparent concentration of analyte expected for blank samples and the lowest concentration likely to be detected and distinguished from a blank sample respectively. The smallest detectable difference (SDD) is the smallest variance of measurement required to deem two measurements distinct. The LoB and LoD were determined as discussed by Armbruster and Pry (2008) from Clinical and Laboratory Standards Institute (2004), while the SDD as outlined by Kropmans et al. (1999) based on McNemar (1969). Calculations used the below equations where ‘*B*’, ‘*SD*’ and ‘*SE*’ denote concentration readings for 60 blank methanol samples taken from the same plate, standard deviation of those readings and standard error of those readings respectively.

$$\text{LoB}\;=\;{\text{mean}}_{B}+1.645\left(SD_{B}\right),$$

$$\text{LoD}\;=\;\text{LoB}\;+\;1.645(SD_{B}),$$

$$\text{SDD}\;=\;1.96\left(\sqrt{2(SE)}\right).$$

### 2.6 Statistical analysis

All statistical analyses were performed using R version 3.5.3 (R Core Team, 2020). To compare the macronutrient content of the species analysed, multivariate linear models (MLMs) were fitted using the ‘manylm’ function of the mvabund package (Wang et al., 2012). Ternary plots were produced via ‘ggtern’ (Hamilton & Ferry, 2018) and ‘ggplot2’ (Wickham, 2016).

## 3 RESULTS

### 3.1 Calculation of methodological boundaries

Macronutrient Extraction and Determination from Invertebrates successfully determined protein, carbohydrate and lipid content directly in parallel from a range of invertebrates, with a turnaround time from sample to data of 3 days and at a cost of ~$1.35 per sample using standard laboratory equipment (heating block, shaker, bead beater and plate reader). Limits of detection using normal standard curve concentrations, reagent ratios and solvent volumes facilitate analysis of all but carbohydrate in an invertebrate as small as an aphid, although differences between single aphids may not be accurately detectable (Table 2).

**TABLE 2:** Limit of blank (LoB), limit of detection (LoD) and smallest detectable difference (SDD) calculated from repeat methanol blanks, and single aphid macronutrient content (mean ± *SD*)

	Protein (mg/ml)	Carbohydrate (mg/ml)	Lipid (mg/ml)
LoB	0.067	0.065	0.006
LoD	0.133	0.130	0.011
SDD	0.321	0.317	0.093
Single aphid content	0.17 ± 0.09	<0.01 ± <0.01	0.17 ± 0.03

Macronutrient Extraction and Determination from Invertebrates successfully detected significant differences in proportional macronutrient content between species (MLM: *F* = 38.91, *p* = 0.002; Table 3).

**TABLE 3:** Macronutrients determined from each of the five species expressed as absolute macronutrient mass (mass mg; mean ± *SD*), percentage of body mass (%mass; mean ± *SD*) and percentage of total macronutrient mass (%macronutrients; mean ± *SD*). Values were calculated from eight individuals of each species (except body mass‐related values for one specimen of *Folsomia candida*). The presented protein values were determined via the Lowry assay (Table S1)

Species	Carbohydrate			Lipid			Protein		
	Mass (mg)	%mass	%macronutrients	Mass (mg)	%mass	%macronutrients	Mass (mg)	%mass	%macronutrients
*Acheta domesticus*	0.05 ± 0.01	0.23 ± 0.02	0.54 ± 0.07	1.62 ± 0.41	7.41 ± 1.16	17.49 ± 3.53	8.05 ± 3.30	35.35 ± 5.15	81.97 ± 3.55
*Blattella germanica*	0.07 ± 0.06	0.29 ± 0.20	0.67 ± 0.55	1.71 ± 0.48	7.76 ± 2.16	17.15 ± 8.60	10.25 ± 4.56	48.34 ± 26.18	82.18 ± 8.86
*Folsomia candida*	0.01 ± <0.01	0.43 ± 0.22	3.90 ± 2.61	0.05 ± 0.02	5.88 ± 3.70	35.35 ± 17.07	0.010 ± 0.05	8.75 ± 3.98	60.76 ± 15.68
*Metopolophium dirhodum*	<0.01 ± <0.01	0.14 ± 0.05	1.33 ± 0.42	0.17 ± 0.03	5.66 ± 1.32	51.67 ± 12.12	0.17 ± 0.09	5.62 ± 2.63	47.01 ± 12.26
*Tenebrio molitor*	0.18 ± 0.18	0.43 ± 0.38	1.16 ± 0.91	2.08 ± 0.41	7.31 ± 2.67	19.93 ± 6.76	9.99 ± 5.18	28.74 ± 6.50	78.91 ± 6.16

The gravimetric lipid mass and exoskeletal mass were determined for the three focal species for which body mass could be accurately measured (Table 4).

**TABLE 4:** Body mass, exoskeletal mass and gravimetric lipid mass for *Acheta domesticus*, *Blattella germanica* and *Tenebrio molitor*. Body mass and gravimetric lipid mass values were calculated from eight individuals of each species (seven for *A. domesticus* and *B. germanica* gravimetric lipid mass), while exoskeletal mass values were calculated from a separate five individuals of each species

Species	Body mass (mg)	Exoskeletal mass, % body mass	Gravimetric lipid mass, % body mass
*Acheta domesticus*	22.20 ± 5.83	13.03 ± 1.93	16.75 ± 6.20
*Blattella germanica*	22.53 ± 4.96	19.75 ± 1.44	12.78 ± 8.65
*Tenebrio molitor*	36.20 ± 22.30	14.34 ± 1.85	28.81 ± 6.06

## 4 DISCUSSION

Macronutrient Extraction and Determination from Invertebrates (MEDI) successfully measured macronutrient content directly and rapidly from the same macroinvertebrate, even as small as a single aphid or collembolan, or as large as a tenebrionid larva or German cockroach. Aphid macronutrient content exceeds the LoDs except for carbohydrate, confirming a sensitivity broadly appropriate for small arthropods and other samples. The relatively low concentration of lipid and carbohydrate estimated in many invertebrate bodies may result in difficulties quantifying at least carbohydrates in such invertebrates (Bryer et al., 2015; Finke, 2005), but the extraction procedure could overcome this by using smaller solvent volumes (e.g. 0.5 ml) to increase the solution concentration, leaving enough material to complete all three assays, or altering the plate incubation times, reagent concentrations and standard concentrations. Directly comparing the macronutrient contents of small invertebrates at an individual level via MEDI could prove difficult without taking such measures given a moderately high SDD relative to the content of the specimens tested. For larger samples, care should be taken to keep readings within the calibration curve; for this, sample dilutions are recommended following an initial test. Increased standard concentrations are not recommended. Prior studies have sometimes used only chloroform, rather than chloroform and methanol, for lipid extraction (Wilder et al., 2013), which can be considered for future applications.

Of the protein assays compared, Lowry was selected as the preferred assay in the case of the specimens tested. While the overall results from the BCA assay were not greatly dissimilar to those of Lowry, the values for German cockroaches regularly exceeded the entire mass of the cockroach, indicating some inaccuracy. This issue may result from German cockroaches storing nitrogen as uric acid in their bodies (Patiño‐Navarrete et al., 2014). Uric acid is known to interfere with the BCA assay, as per the manufacturer notes. In fact, there are many chemicals that can interfere with the BCA assay (Vashist & Dixit, 2011) and indeed most assays. Such inhibitors could be eliminated by introducing a purification step such as trichloroacetic acid protein precipitation, but this is unlikely to be necessary in most cases. Rather than highlighting an optimal assay, this emphasises the importance of selecting assays and standards to best match the context of the work being carried out. The detection of different amino acids by each assay, their consequently differential relevance to protein standards and their variable performance in the presence of inhibitory compounds thus warrants a case‐by‐case consideration of the optimal assay to use, or the averaging of values from a range of assays or standards.

The disparity in colorimetric and gravimetric measurements of lipids could highlight that these assays measure different pools of lipids with the sulfo‐phospho‐vanillin method only measuring unsaturated lipid content while the gravimetric method measures total lipid content. There were inaccuracies in the weighing of these specimens, with one specimen returning a negative mass and two negative gravimetric lipid values (these were thus removed from any calculations relying on these values). The large variability in overall body mass (due to differences in growth stage and possibly body condition) of the tested organisms may have impacted their similarity in macronutrient content. Particularly for the smaller invertebrates, for which body mass measurements are difficult, the proportional content of macronutrient content can be used as an effective proxy for studies concerned with a given taxon's nutritional quality. Alternatively, several specimens can be pooled, as is done in many existing protocols, if only to weigh them together to calculate an average individual mass, or length–mass relationships can be determined from many individuals, but the accuracy, particularly for smaller invertebrates, could be poor. Such pooling, if maintained for assay preparation, could ensure sufficient concentrations to overcome the limits of detection for smaller invertebrates.

Micronutrients were not considered in this protocol, despite their biological importance (Jing et al., 2014), as they do not comprise a single detectable or quantifiable group. Without considering a specific micronutrient, or a subset of them, their quantification can be laborious and, given the expectedly minute content of micronutrients in each invertebrate, detection, much less quantification, of micronutrients may be unfeasible for all but the largest macroinvertebrates without specialised equipment.

## 5 SUMMARY

Macronutrient Extraction and Determination from Invertebrates accurately detects macronutrients for a broad range of potential experimental applications involving invertebrates and other tissues, improving upon existing protocols for macronutrient determination. The protocol is relatively cheap, fast and simple and could present a uniform standard to be used across ecological studies.

## AUTHORS' CONTRIBUTIONS

J.P.C., C.T.M., W.O.C.S., J.R.B., P.O.‐t.W. and I.P.V. conceived the ideas and oversaw the project; J.P.C., S.M.W. and C.T.M. designed the protocol; J.P.C., S.M.W., M.P.T.G.T., R.H., S.O., P.S.M. and R.A.B.‐G. provided samples, and tested and calibrated the protocol; J.P.C. analysed data and led writing of the manuscript. All authors commented upon and contributed to the drafts and approved the final manuscript for publication.

### PEER REVIEW

The peer review history for this article is available at https://publons.com/publon/10.1111/2041‐210X.13551.

## Supporting information

Supplementary Material

## Data Availability

Data archiving: The data used to generate this manuscript are available via Dryad Digital Repository https://doi.org/10.5061/dryad.m905qfv0c (Cuff et al., 2021).
