# Supplementary material for: MEDI: Macronutrient Extraction and Determination from invertebrates, a rapid, cheap and streamlined protocol
Source: Methods Ecol Evol. 2021 Jan 22;12(4):593–601. doi: 10.1111/2041-210X.13551 (PMC8614113; doi:10.1111/2041-210X.13551)
Supplement: Supplementary file 1 — Supplementary Material [file MEE3-12-593-s001.docx]

**Supporting Information**

## Supporting Information 1: Sulfo-phospho-vanillin assay

For determination of lipids, a sulfo-phospho-vanillin method adapted from Cheng *et al*. (2011) was used (Figure 3 main text). This was adapted from a microplate colorimetric assay to estimate invertebrate lipid content, originally described by Van Handel (1985) and considered reliable for small samples (Lu *et al.* 2008). This method determines unsaturated lipid content; for total lipid content, gravimetric methods are the most appropriate option, but difficult without specialised scales for small invertebrates. A standard dilution series was prepared with lard oil diluted with methanol. Samples for lipid analysis comprised the initial supernatant taken after chloroform/methanol extraction. From each sample and standard, three repeats of 50 µl were placed in a heating block at 100 °C for approximately 10 min to evaporate the solvent, after which 10 µl concentrated sulfuric acid was added to each, vortexed and incubated at 100 °C for 10 min. The samples were cooled to room temperature and 240 µl vanillin reagent (1.2 mg dissolved in 0.2 ml hot water and 0.8 ml 85% phosphoric acid) was added and vortexed. After 5 min, 200 µl of each sample and standard were loaded into a 96-well microplate and absorbance at 490 nm was measured.

## Supporting Information 2: Protein assays

For protein determination, two assays were used (Figure 4 main text). First, the Pierce bicinchoninic acid (BCA) protein assay, originally proposed by Smith *et al.* (1985) was used. This method colorimetrically detects a purple product formed by the chelation of two molecules of BCA to a cuprous ion produced via a biuret reaction. A standard dilution series was prepared with bovine serum albumin (BSA) diluted with polished water. Other standards such as IgG can be used and conversion factors exist for any discrepancy between standards; choice of a standard should depend on proximity of the standard to the proteins of the focal species. For each sample and standard, 200 µl of BCA working reagent was prepared by mixing 50 parts BCA reagent A with 1 part BCA reagent B (Fisher Scientific). Of each sample and standard, three repeats of 25 µl were added to a 96-well microplate with 200 µl of the working reagent; this was mixed in a thermo-mixer at room temperature for 30 s at 450 rpm before incubation at 37 °C for 10 min. The plate was cooled to room temperature and the absorbance at 562 nm measured.

Additionally, the Pierce modified Lowry protein assay, originally proposed by Lowry *et al.* (1951) was used. This method colorimetrically detects heteropolymolybdenum Blue, a molecule formed by the reaction of the Folin-Ciocalteu reagent with Cu^+^ produced by peptide bond oxidation. The same standard dilution series was prepared as for the BCA assay. From each standard and sample, three replicates of 40 µL were mixed with 200 µL of the Modified Lowry Reagent and incubated at room temperature for 10 minutes. To each well, 20 µl of 1X (1N) Folin-Ciocalteu reagent was added, incubated at room temperature for 30 minutes and absorbance at 750 nm measured.

## Supporting Information 3: Anthrone assay

For carbohydrate determination, the anthrone method, originally proposed by Dreywood (1946), was used (Figure 5 main text). This method colorimetrically detects a blue-green complex formed by condensation by anthrone with furfural produced by hydrolysis of carbohydrates with acid. The method is best applied to sugars; if complex carbohydrates (e.g. trehalose, glycogen) are of interest, specific assays can be incorporated into the protocol. A standard dilution series was prepared with corn starch diluted with polished water. The anthrone reagent was prepared by dissolving 1 mg of anthrone in 1 ml of concentrated (>95 %) H_2_SO_4_. From each standard and sample, three repeats of 40 µl were added to a 96-well microplate and each mixed with 160 µl anthrone reagent before mixing in a thermo-mixer at room temperature for 30 s at 450 rpm before. The plate was incubated at 92 °C for 10 min and cooled to room temperature. Absorbance at 620 nm was measured.

**Table S1: Protein determined via the two trialled assays from each of the five species expressed as absolute protein mass (mass mg), percentage of body mass (%mass) and percentage of total macronutrient mass (%macronutrients). Values were calculated from eight individuals of each species. Values for %mass of *F. candida* and *M. dirhodum* are absent due to inaccuracies associated with determining the body mass of such small specimens.**

| **Species** | **BCA** | | | **Lowry** | | |
| --- | --- | --- | --- | --- | --- | --- |
|  | **Mass (mg)** | **%mass** | **%macronutrients** | **Mass (mg)** | **%mass** | **%macronutrients** |
| *Acheta domesticus* | 11.02  ± 2.60 | 50.30  ± 6.06 | 86.73 ± 2.05 | 8.05  ± 3.30 | 35.35  ± 5.15 | 81.97 ± 3.55 |
| *Blatella germanica* | 22.76  ± 6.99 | 111.89 ± 55.42 | 91.65 ± 4.61 | 10.25  ± 4.56 | 48.34  ± 26.18 | 82.18 ± 8.86 |
| *Folsomia candida* | 0.20  ± 0.10 | NA | 76.26 ± 10.15 | 0.010  ± 0.05 | NA | 60.76 ± 15.68 |
| *Metopolophium dirhodum* | 0.37  ± 0.07 | NA | 67.33 ± 7.25 | 0.17  ± 0.09 | NA | 47.01 ± 12.26 |
| *Tenebrio molitor* | 12.36  ± 2.22 | 42.63  ± 14.39 | 84.60 ± 2.04 | 9.99  ± 5.18 | 28.74  ± 6.50 | 78.91 ± 6.16 |

## **References**

Cheng, Y.-S., Zheng, Y. and VanderGheynst, J.S. (2011). Rapid quantitative analysis of lipids using a colorimetric method in a microplate format. *Lipids* **46**:95–103.

Dreywood, R. (1946). Qualitative test for carbohydrate material. *Industrial and Engineering Chemistry* **18**:499.

Van Handel, E. (1985). Rapid determination of total lipids in mosquitoes. *Journal of the American Mosquito Control Association* **1**:302–304.

Lowry, O.H., Rosebrough, N.J., Farr, A.L. and Randall, R.J. (1951). Protein measurement with the Folin phenol reagent. *Journal of Biological Chemistry* **193**:265–75.

Lu, Y., Ludsin, S.A., Fanslow, D.L. and Pothoven, S.A. (2008). Comparison of three microquantity techniques for measuring total lipids in fish. *Canadian Journal of Fisheries and Aquatic Sciences* **65**:2233–2241.

Smith, P.K., Krohn, R.I., Hermanson, G.T., Mallia, A.K., Gartner, F.H., Provenzano, M.D., Fujimoto, E.K., *et al.* (1985). Measuring of protein using bicinchoninic acid. *Analytical Biochemistry* **150**:76–85.
